# The Psychological Impacts of Pill Dysphagia: A Mixed Methods Study

**DOI:** 10.1007/s00455-024-10703-4

**Published:** 2024-04-18

**Authors:** Rowan Adams, Dimity A Crisp, Jackson Thomas

**Affiliations:** Faculty of Health, University of Canberra, Canberra, ACT 2617 Australia

**Keywords:** Swallowing, Pills, Wellbeing, Older adults, Australian

## Abstract

Pill dysphagia is a common problem amongst older adults, with significant health consequences. Previous research has found that dysphagia can negatively affect an individuals mental health and wellbeing. However, this research has not been extended to pill-specific dysphagia, which presents distinct differences from the challenges posed by swallowing food and liquids. These differences extend to causes, demographics, and physical health ramifications. This study aimed to address this gap in the literature by investigating the effects of pill dysphagia on the wellbeing of older adults. A community sample of 132 Australians aged 65–97 years completed a survey about their wellbeing and difficulty swallowing pills. Thirty-one participants who met the criteria for pill dysphagia completed further open-ended questions detailing the effects of pill dysphagia and how they manage it. Analyses of the quantitative data indicated that difficulty swallowing pills was unrelated to negative affect but negatively related to positive affect, life satisfaction, and eudemonic wellbeing. Supplementary analyses controlling for health-related variables found no significant relationships between difficulty swallowing pills and wellbeing. Responses to the open-ended questions revealed a range of physical, psychological, and practical impacts of pill dysphagia, and successful and unsuccessful methods used to assist in swallowing pills. The findings partially support the hypothesised effects of pill dysphagia on wellbeing. However, further research is required to establish if more severe pill dysphagia predicts wellbeing over and above self-rated health. Future interventions should incorporate wellbeing promotion strategies for older adults with pill dysphagia.

## Introduction

Solid oral dosage forms (pills) are the most common, preferred, and convenient form of medication administration [1, 2]. Older adults are particularly reliant on pills. In Australia, 84% of adults aged 70 years and over take at least one prescription medicine daily, and 45% take five or more [3]. Dysphagia, characterised by difficulty swallowing, affects the intake of food, liquid, and oral medications. Dysphagia can result from a range of aetiologies; however, it is most prevalent in older adults due to ageing-related diseases like stroke and neurodegenerative conditions [4]. Around 30% of community-dwelling older adults and 59% of aged care residents are affected by dysphagia [5]. Pill-specific dysphagia is experienced by around 14% of community-dwelling older adults and approximately 10–30% of aged care residents [6, 7]. Difficulty in swallowing pills impacts adherence, leading to morbidity and mortality risks [8]. Approximately 10% of community pharmacy customers and a quarter of aged care facility residents modify their medications [9, 10]. Up to one-third of these modifications are inappropriate [10]. Modifying pills to ease swallowing poses dangers, and can lead to medication intake issues, choking, and even death [6, 7, 11]. The risks associated with difficulty swallowing and the modification of pills to ease swallowing further underscore the health implications of dysphagia in older adults. The detrimental effects of pill dysphagia may also extend to an individual’s wellbeing; however, there is limited research in this area.

Wellbeing can be conceptualised in terms of hedonic and eudemonic components. The hedonic tradition equates wellbeing with subjective happiness [12] and consists of positive emotion, negative emotion, and cognitive evaluations of one’s life [13]. Optimal wellbeing is conceptualised as having positive affect (e.g., alertness, enthusiasm), minimal negative affect (e.g., calmness, serenity), and a high level of overall satisfaction with life [14, 15]. The eudemonic perspective supports that wellbeing also involves assessment of positive functioning and personal fulfillment [16], and includes components such as autonomy, environmental mastery, personal growth, self-acceptance, positive relatedness and purpose in life [17]. The multidimensional structure of wellbeing as a combination of hedonic and eudemonic domains has been demonstrated in older adults [18]. While levels of life satisfaction, affective wellbeing, and eudemonic wellbeing have been shown to vary with age [19], maintaining high hedonic and eudemonic wellbeing in old age can have protective effects on health and mortality [20–22].

Physical illnesses, many of which become more prevalent with age, are known to negatively impact all dimensions of wellbeing [19]; however, the specific effects of pill dysphagia on mental health and wellbeing are relatively unknown. Research has shown that general dysphagia is linked to higher stress, depression, anxiety, and decreased emotional quality of life [23, 24]. Dysphagic patients report low levels of purpose in life, comparable to severe cancer patients [25]. Qualitative accounts reveal feelings of loss of autonomy and environmental mastery, aspects of eudemonic wellbeing [26, 27]. Dysphagia’s association with poor wellbeing is likely linked to difficulty swallowing food, and consequent dietary restrictions, which have both psychological and social impacts [28–30]. However, as pill dysphagia does not involve difficulty swallowing food, its effects on wellbeing may differ from general dysphagia. Ohrnberger et al.’s health framework [31] suggests that physical health influences mental health through direct and indirect pathways. Pill dysphagia may lower life satisfaction, affect emotions, and reduce positive psychological functioning as a result of medication non-adherence, which is known to affect quality of life [32, 33], or through instilling a fear of choking [34]. However, the relationship between pill dysphagia and poorer wellbeing has not yet been investigated. Although the negative impact of dysphagia on wellbeing is well documented, there is a clear need for more research to explore how difficulty swallowing pills influences wellbeing.

This study examined the impact of difficulty swallowing pills on hedonic and eudemonic wellbeing in older adults. Based on existing research [23, 25, 35], we hypothesised that greater severity of pill dysphagia would be associated with: (1) higher negative affect; (2) lower positive affect; (3) lower life satisfaction; and (4) overall lower eudemonic wellbeing. Additionally, this study qualitatively explored the overall impact of pill dysphagia on individuals’ perceptions of wellbeing, as well as their strategies to cope with the impact.

## Materials and Methods

### Participants and Procedure

Participants were Australian residents (*n* = 132) aged 65 to 97 years (*M* = 73, *SD* = 6.4). 59% of participants were female, reflecting a small overrepresentation in comparison to the older Australian population (53% female) [36]. Participants completed an online survey administered using Qualtrics survey software between June and August 2022. Recruitment reflected a convenience sample approach and occurred through advertising at retirement communities, education and healthcare organisations for older adults, and via Facebook. Ethical approval was obtained from the University of Canberra Human Research Ethics Committee (Project ID 11664).

### Measures

The survey included validated measures to assess health, wellbeing, and pill dysphagia. In addition to the measures detailed below, participants indicated if they had any health conditions that are typically comorbid with dysphagia. Participants answered yes/no to the following conditions: Parkinson’s Disease, head or neck cancer, Stroke, Alzheimer’s Disease, Multiple Sclerosis, Pneumonia, Gastro-oesophageal reflux disease, Motor Neurone Disease, traumatic brain injury, and an ‘other’ option for any other condition that affects their ability to swallow. Responses were summed to create a scale reflecting number of conditions an individual had. Participants rated their overall health on a scale from 1 (*poor*) to 5 (*excellent*). They were also asked how often they take pills, with answers on a scale from 1 (*once a week or less*) to 4 (*multiple times a day*). Pilot testing of the survey was conducted for clarity of wording before administration. Scale measures reported high internal consistency in the present sample (see Table 1).

**Table 1:** Descriptive statistics and bivariate correlations between variables

Variable	α	M	SD	PILL-5	LS	PA	NA	EW
1. PILL-5	0.78	3.39	3.57	—				
2. Life Satisfaction	0.90	24.74	6.80	− 0.29***	—			
3. Positive Affect	0.91	31.16	7.52	− 0.23**	0.49***	—		
4. Negative Affect	0.91	15.27	6.06	0.17*	− 0.53***	− 0.24**	—	
5. Eudemonic Wellbeing	0.91	39.71	9.79	− 0.29***	0.76***	0.66***	− 0.52***	—

*Notes*: α = internal consistency for scale; M = mean, SD = standard deviation, LS = life satisfaction, PA = positive affect, NA = negative affect, EW = Eudemonic wellbeing; * *p* < .05, ** *p* < .01, *** *p* < .001

### Wellbeing

The following scales were used to measure aspects of wellbeing, each of which had been validated and used with older adult populations [37, 38]. Life satisfaction was assessed using the 5-item Satisfaction with Life Scale (SWLS) [39]. Respondents reported the extent to which they agreed with each statement on a 7-point Likert scale ranging from 1 (*strongly disagree*) to 7 (*strongly agree*). Total scores ranged from 7 to 35, with higher scores reflecting greater life satisfaction.

The affective component of hedonic wellbeing was measured using the 20-item Positive and Negative Affect Schedule (PANAS) [15]. The PANAS comprises two 10-item subscales capturing the respondent’s level of positive affect and negative affect. Participants reported the extent to which they have felt each emotion over the past week, with responses recorded using a 5-point Likert scale ranging from 1 (*very slightly or not at all*) to 5 (*extremely*). Total scores for each subscale range from 10 to 50, with higher scores representing higher levels of positive or negative affect.

Eudemonic wellbeing was assessed using the Control, Autonomy, Self-Realisation and Pleasure (CASP-19) [40] scale. The CASP-19 was explicitly developed for older adults and consists of 19 items measuring control, autonomy, self-realisation and pleasure. Participants were asked how they have felt over the past week, with responses recorded using a 4-point Likert scale ranging from 0 (*never*) to 3 (*often*). Total scores range from 0 to 57, with higher scores reflecting greater eudemonic wellbeing.

### Pill Dysphagia

The degree of difficulty swallowing pills was determined by self-report, using the PILL-5 scale [41]. The PILL-5 consists of five indicators of pill dysphagia, such as “Pills stick in my throat.”. Responses are measured on a 5-point Likert scale ranging from 0 (*never*) to 4 (*always*). Total scores range from 0 to 20, with higher scores reflecting greater severity, and scores of 6 or more indicating pill dysphagia [41]. The scale has been validated for use with dysphagia patients and healthy individuals [41].

Participants who scored 6 or more on the PILL-5 completed three open-ended questions about their experiences with pill dysphagia. These questions were: “In what way does your difficulty swallowing pills affect your life?”, “Does your difficulty swallowing pills affect how you feel? In what way?”, and “Are there any methods you use to make swallowing pills easier to deal with?”.

## Results

### Data Analysis

Quantitative data was analysed using Jamovi version 2.3.16. Bivariate correlations were calculated to examine relationships between variables. Generalised linear models (GLMs) were then conducted to examine the influence of difficulty swallowing pills on the outcome variables of life satisfaction, positive affect, negative affect, and eudemonic wellbeing. As gender is known to be associated with wellbeing [42, 43], it was controlled for in all analyses. A gamma distribution was used to accommodate a positive skew in negative affect [44]. Due to the correlations between the outcome variables and self-rated health, number of health conditions, and frequency of taking pills, supplementary analyses were conducted controlling for these variables.

Responses to the open-ended questions were analysed using inductive thematic analysis, following Braun and Clark’s [45] guidelines. Initial codes were clustered into potential themes, followed by iterative review and refinement to ensure coherence with the questions. Frequency counts quantified each theme’s occurrence. Selected data extracts were then employed to illustrate participants’ experiences related to pill dysphagia.

### Sample Characteristics

A high frequency of taking pills was reported in the sample with 92% (*n* = 121) of the sample reporting that they take pills at least once a day (62% reported multiple times a day). 25% (*n* = 33) of participants scored 6 or over on the PILL-5, suggesting that they suffered from pill dysphagia. 3% (*n* = 4) of participants scored 11 or over on the PILL-5, placing them in the moderate to severe pill dysphagia category [41]. 47% (*n* = 62) of participants reported at least one health condition frequently comorbid with dysphagia. In reporting self-rated health, on average, participants considered themselves to be fairly healthy (*M* = 3.71, *SD* = 0.90). Descriptive statistics for wellbeing indicators are presented in Table 1 along with bivariate correlations with the PILL-5 score. Participants reported moderate levels of satisfaction with life, positive affect and eudemonic wellbeing, and low levels of negative affect. Bivariate correlations between variables indicated greater difficulty swallowing pills was weakly associated with lower life satisfaction, eudemonic wellbeing, and positive affect, and higher levels of negative affect.

### Difficulty Swallowing Pills as a Predictor of Wellbeing

A series of GLMs were conducted to assess the role of difficulty swallowing pills in predicting each wellbeing outcome, controlling for gender. A summary of the results is presented in Table 2. PILL-5 score was significantly negatively related to life satisfaction, positive affect, and eudemonic wellbeing, with the models accounting for 6.3% (*z*(1)=-3.01, *p* = .003), 4.4% (*z*(1)=-2.43,*p* = .017) and 7.1% (*z*(1)=-3.16, *p* = .002) of the variance in each of these outcome variables respectively. PILL-5 score was not found to be a significant predictor of negative affect.

**Table 2:** Generalised linear models predicting wellbeing

Outcome	Predictor	Estimate	SE	95% CI		
				Lower	Upper	Exp(B)
Life Satisfaction	Gender	-1.73	1.19	-4.07	0.61	0.18
	PILL-5 Score	-0.50**	0.17	-0.82	-0.17	0.61
Positive Affect	Gender	-0.95	1.35	-3.60	1.70	0.39
	PILL-5 Score	-0.45*	0.19	-0.82	-0.09	0.63
Negative Affect	Gender	0.08	0.07	-0.06	0.22	1.09
	PILL-5 Score	0.02	0.01	-0.00	0.04	1.02
Eudemonic Wellbeing	Gender	-0.96	1.73	-4.36	2.44	0.38
	PILL-5 Score	-0.76**	0.24	-1.23	-0.29	0.47

*Notes*: * *p* < .05, ** *p* < .01

Considering the established link between physical health, swallowing difficulties, and wellbeing [46, 47], we conducted additional analyses by factoring in self-rated health, pill frequency, and health conditions. This adjustment negated all significant associations between PILL-5 score and wellbeing. Notably, self-rated health emerged as the sole predictor of wellbeing outcomes. The results of these analyses are presented in Table 3.

**Table 3:** Hierarchical generalised linear models predicting wellbeing

Outcome			Predictor	Estimate	SE	95% CI		
						Lower	Upper	Exp(B)
Life Satisfaction	Model 1		Gender	-2.31*	1.05	-4.38	-0.25	0.10
			Pill Frequency	-0.47	0.61	-1.66	0.73	0.63
			Health Conditions	-0.90	0.72	-2.31	0.52	0.41
			Self-rated Health	3.41***	0.60	2.24	4.58	30.20
	Model 2		Gender	-1.90	1.07	-4.00	0.21	0.15
			Pill Frequency	-0.37	0.61	-1.56	0.82	0.69
			Health Conditions	-0.62	0.73	-2.06	0.82	0.54
			Self-rated Health	3.28***	0.60	2.10	4.45	26.44
			PILL-5 Score	-0.27	0.16	-0.57	0.04	0.77
Positive Affect	Model 1		Gender	-1.70	1.23	-4.11	0.71	0.18
			Pill Frequency	0.48	0.71	-0.91	1.88	1.62
			Health Conditions	-0.32	0.84	-1.97	1.32	0.72
			Self-rated Health	3.73***	0.70	2.36	5.09	41.64
	Model 2		Gender	-1.26	1.25	-3.72	1.20	0.28
			Pill Frequency	0.58	0.71	-0.81	1.98	1.79
			Health Conditions	-0.03	0.86	-1.71	1.65	0.97
			Self-rated Health	3.59***	0.70	2.22	4.96	36.15
			PILL-5 Score	-0.28	0.18	-0.64	0.07	0.75
Negative Affect	Model 1		Gender	0.12	0.07	-0.01	0.25	1.13
			Pill Frequency	0.02	0.04	-0.06	0.10	1.02
			Health Conditions	0.02	0.05	-0.07	0.11	1.02
			Self-rated Health	-0.11**	0.04	-0.18	-0.03	0.90
	Model 2		Gender	1.31	1.09	-0.83	3.45	3.71
			Pill Frequency	0.25	0.62	-0.96	1.46	1.28
			Health Conditions	0.11	0.74	-1.34	1.57	1.12
			Self-rated Health	-1.63**	0.61	-2.82	-0.44	0.20
			PILL-5 Score	0.14	0.16	-0.17	0.45	1.15
Eudemonic Wellbeing	Model 1		Gender	-1.68	1.35	-4.34	0.97	0.19
			Pill Frequency	-0.07	0.78	-1.60	1.47	0.94
			Health Conditions	-1.99	0.93	-3.80	-0.18	0.14
			Self-rated Health	6.46***	0.77	4.95	7.96	637.89
	Model 2		Gender	-1.19	1.38	-3.89	1.52	0.31
			Pill Frequency	0.05	0.78	-1.49	1.58	1.05
			Health Conditions	-1.66	0.94	-3.51	0.19	0.19
			Self-rated Health	6.30***	0.77	4.79	7.81	544.32
			PILL-5 Score	-0.32	0.20	-0.71	0.07	0.73

*Notes*: ** *p* < .01, *** *p* < .001

### Experiences with Pill Dysphagia: Qualitative Analysis

Of the 33 participants who scored 6 or more on the PILL-5, 31 responded to open-ended questions about their experiences with pill dysphagia. Thematic analysis identified several themes relating to the way in which difficulty swallowing pills affects their life, makes them feel, and how they manage it (see Table 4).

**Table 4:** Summary of themes and frequency of responses

Question	N	Theme	%	n
In what way does your difficulty swallowing pills affect your life?	29	Physical health effects	69.0	20
		Practical difficulties and life disruptions	34.5	10
		Psychological impacts	31.0	9
		Life is unaffected	17.2	5
Does your difficulty swallowing pills affect how you feel? In what way?	30	Psychological effects	66.7	20
		Physical effects	16.7	5
		Practical difficulties and life disruptions	13.3	4
		It does not affect how I feel	20.0	6
Are there any methods you use to make swallowing pills easier to deal with? If so, what are they?	31	Pill-swallowing aids	67.7	21
		Modifications	41.9	13
		Experiences with failed methods	16.1	5
		Adaption and barriers to adapting	16.1	5
		Compensatory techniques	12.9	4
		Relaxation techniques	12.9	4

### The Effects of Pill Dysphagia

In response to “In what way does your difficulty swallowing pills affect your life?”, the most prevalent theme, reported by 69% of respondents (*n* = 20), was related to the *physical health effects* of pill dysphagia. Participants experienced adverse effects when attempting to swallow pills, ranging from discomfort, to choking and vomiting. Pill dysphagia imposed restrictions on the medications that participants could take, with one participant stating that they were unable to treat an unrelated health condition because they could not swallow the required medication. Other participants were forced to modify their medications, which could also have adverse effects, “I worry about going to the hospital and having them cut oils [sic.] up which gives them sharp edges which makes my throat swell up”.

*Practical difficulties* and life disruptions were reported by approximately 35% of respondents (*n* = 10). These included effects on routines and social life. Participants discussed needing to be at home at the same time each day to take their pills, as modification requirements and the possibility of adverse reactions made them unwilling to take pills elsewhere. Similarly, some participants only took pills in private due to embarrassment about their difficulty swallowing. One participant, however, expressed preferring to take pills in the company of others, due to their fear of choking.

Almost one-third of respondents (31%, *n* = 9) indicated *psychological impacts* associated with difficulty swallowing pills, presenting as anxiety and fear related to swallowing pills. Participants’ fear of choking was particularly apparent. One participant wrote, “I have previously choked and this has left me with a fear of dying from a pill caught in my throat”. Another described choking as a “frightening” experience. Other participants were anxious about how pill dysphagia may affect their ability to treat or manage future illnesses.

While most respondents agreed that pill dysphagia affected their lives, five disagreed, as reflected by theme four: *life is unaffected*. Some elaborated that pill dysphagia was a rare occurrence or minor annoyance, while others reported no effect at all, indicating a variable impact on patients’ lives.

### How does Pill Dysphagia make you Feel?

In the response to the question “Does your difficulty swallowing pills affect how you feel? In what way?”, the themes identified from participant responses largely mirrored those for question one. However, the content and prevalence of these themes differed. In response to this question, two-thirds of participants (67%, *n* = 20) indicated *psychological effects* of pill dysphagia. Anxiety and fear were again common topics. Participants were worried about choking and about the possibility of modified medications impacting their health. Frustration was also reported. There was a sense among some participants that pill dysphagia compounded other stressors in their lives. One participant captured this in relation to his degenerative muscle disease, “It’s another reminder that I have an incurable degenerative disease …. It also sometimes makes it hard for me to take the pills that at least add some comfort to my life under my disease conditions”. Pill dysphagia also negatively affected how some participants saw themselves. For example, one explained, “I feel stupid that I have difficulty swallowing large pills when others seem to do it easily”, while another wrote, “I feel foolish”. Although the emotional and self-conceptual effects of pill dysphagia were overwhelmingly negative, one participant had a more positive perspective. They wrote: “[It] can be very pleasing if the pills go down easy”. This sign of positivity suggests that some pill dysphagia patients can find satisfaction in small successes.

Five participants reflected on *physical effects* by indicating that pill dysphagia affected how they felt physically. For some, pill dysphagia exacerbated their other health conditions, while others experienced discomfort, nausea, or reflux.

A further four participants indicated *practical difficulties* and life disruptions. For some, pill dysphagia affected their routine or their medication options, echoing some of the responses to question one. One participant wrote about how the difficulty finding appropriate pills made her feeling inadequate. Another described how the physical and emotional effects of pill dysphagia caused her to avoid certain activities, “The feeling of chest discomfort [from swallowing pills] also makes me a bit anxious so I avoid doing anything strenuous …. Sometimes I worry a bit about driving in case something happens”.

Finally, 6 participants indicated that it *did not affect how they felt*. These participants, however, reported relatively low PILL-5 scores (between 6 and 8), and also largely indicated that they experienced mild life disruptions in the previous question.

### Strategies for Managing Pill Dysphagia

In their responses to question three, “Are there any methods you use to make swallowing pills easier to deal with? If so, what are they?”, all respondents reported at least one method. Six themes were drawn from the responses, the most common of which were pill-swallowing *aids and modifications*. Approximately 68% of respondents (*n* = 21) took pills with fluids or food to help them swallow. The modification of pills was reported by 42% (*n* = 13) of respondents. Most either crushed or cut their pills, although one participant chewed them.

Attempting to make pills easier to swallow was not always successful, as reflected by the theme: *experiences with failed methods* reported by 16% (*n* = 5) of respondents. Some participants recounted injurious modification attempts, “I tried opening [the capsule] and mixing the contents with jam … but after [doing that] I had bad reflux so I haven’t done that again”. Others could only swallow pills after multiple unsuccessful attempts.

For other participants (16%, *n* = 5), management of pill dysphagia involved *adaption* and choosing easy-to-swallow pills. Where possible, participants selected small pills, coated pills, or those that could be safely modified. However, a lack of options meant that many participants were required to take unmodifiable or difficult-to-swallow pills.

Finally, several participants described using *compensatory* (13%, *n* = 4), *or relaxation techniques* (13%, *n* = 4) to help them swallow pills. Various compensatory techniques were used, including head adjustments, clearing the throat before swallowing, and placing pills on the back of the tongue. In contrast, the types of relaxation techniques used did not vary. Methods appeared to be informal; participants wrote that they would “Try to relax”. Or, as one participant stated, “Try not to panic”.

## Discussion

This study explored individual’s perception of the impact of pill dysphagia on their wellbeing. After adjusting for gender, our hypotheses were partially supported, such that greater difficulty swallowing pills was associated with lower positive affect, life satisfaction, and eudemonic wellbeing. However, difficulty swallowing pills was unrelated to negative affect. Notably, pill dysphagia had the most significant unique contribution to eudemonic wellbeing variance (7%), followed by life satisfaction (6%) and positive affect (4%), though these contributions were relatively small [48]. Once we controlled for self-rated health, number of health conditions, and frequency of taking pills, the effect of pill dysphagia on wellbeing was no longer significant.

In contrast to the quantitative findings, the qualitative analysis uncovered participants’ personal narratives about how pill dysphagia profoundly affected their overall wellbeing and broader quality of life. Notably, participants frequently expressed experiencing negative emotions. Additionally, participants reported facing adverse physical and health repercussions linked to pill dysphagia and practical challenges in managing the condition. The qualitative data also sheds light strategies participants adopted to cope with swallowing difficulty, offering valuable suggestions to address the condition’s impact on their wellbeing.

### The Relationship between Pill Dysphagia and Wellbeing

Pill dysphagia was significantly negatively associated with positive affect, life satisfaction, and eudemonic wellbeing in the primary analyses. These results are consistent with most previous research focusing on the broader diagnosis of dysphagia, involving difficulty swallowing food, liquids, and saliva, as well as medications, which has shown dysphagia to negatively impact aspects of eudemonic wellbeing [25, 26, 29], and has detrimental effects on mental health and quality of life [24, 49]. Responses to the open-ended questions indicate the pathways through which eudemonic wellbeing, positive affect, and life satisfaction may be affected. Principally, participants with pill dysphagia may experience lower eudemonic wellbeing due to a percieved loss of autonomy and lack of control over their health and routine. Pill dysphagia also places potential limitations on an individual’s social life and activities, which can, in turn, decrease life satisfaction and limit opportunities to experience positive affect [50]. This is consistent with Ohrnberger’s health framework [31], wherein lifestyle and social capital partially mediate the relationship between physical and mental health.

The hypothesis that pill dysphagia would predict higher levels of negative affect was not supported, contrasting results seen with general dysphagia [23, 24, 51]. However, experiences of negative affect resulting from difficulty swallowing pills were reported in response to the open-ended questions. Almost half of the participants expressed fear and anxiety related to choking on their medications, and the impact of this on their future health.

As most respondents reported mild to moderate pill dysphagia, it is possible that, although noteworthy for participants, these negative impacts may not have been severe or regular enough to significantly impact negative affect scores on the PANAS. In our sample, only 12% of those meeting criteria indicative of pill dysphagia reported as moderate to severe. This contrasts most previous studies on wellbeing in dysphagia which have included a greater proportion of participants with moderate and severe symptoms [47, 52]. Evidence supports that greater severity of general dysphagia is associated with greater anxiety and depression and lower emotional quality of life [23, 52–55], and has suggested that mild dysphagia may have a negligible impact on mental health outcomes. Whether this pattern is relevant to pill-specific dysphagia and broader wellbeing remains unknown. However, if there is an association between more severe pill dysphagia and lower wellbeing, the low prevalence of severe pill-swallowing difficulties in the current sample may explain the non-significant results.

The qualitative component of the study also supports the assumption that the results were influenced by low pill dysphagia severity. Participants with more severe PILL-5 scores tended to describe more impacts, such as feelings of incompetence and fear of choking. In contrast, participants who scored lower on the PILL-5 reported experiencing minimal or no effects of pill dysphagia. Further research drawing from a population with greater difficulty swallowing pills is necessary to confirm if individuals with severe pill dysphagia experience lower hedonic and eudemonic wellbeing.

After controlling for health-related variables, self-rated health predicted wellbeing indicators over and above difficulty swallowing pills. This is not surprising given that older adults with pill dysphagia, particularly mild to moderate pill dysphagia, are likely to have comorbidities that influence their perceived health and wellbeing more significantly. Although better self-rated health has been associated with higher wellbeing and lower swallowing difficulty [46, 47], previous dysphagia research has failed to control for self-rated health or related factors [35, 56]. Consequently, the results of these studies could have also been confounded by the wellbeing impact of diseases that underlie dysphagia.

### Managing Pill Dysphagia

Examination of the responses to the open-ended questions offers implications for managing pill dysphagia. One-third of participants with pill dysphagia recounted experiences with barriers or failures to manage their difficulty swallowing. Multiple participants felt that easy-to-swallow formulations lacked availability. In addition, over one-third of respondents reported modifying their pills, some of whom had experienced adverse health effects from doing so. These results demonstrate that patients struggle to manage pill dysphagia effectively. However, it is unknown if participants had ever discussed these difficulties with a healthcare professional (including a pharmacist) to establish if alternative options were available to them. There remains a clear need for medication options or pill-swallowing aids that are easier to swallow, as well as education of patients regarding the potential risks of inappropriate modifications [57]. Moreover, interventions to improve swallowing functioning may be beneficial. Future research may seek to examine community literacy in this area.

Most techniques that participants reported using to assist swallowing, namely modifications, compensatory strategies, and pill-swallowing aids, are consistent with those reported by patients previously [58, 59]. The use of relaxation, however, has not been reported by participants in previous studies. Relaxation techniques have been successfully incorporated into treating pill aversion in children and pregnant women [60, 61]. Given the large proportion of respondents who reported fear and anxiety associated with their pill dysphagia, psychological treatments may also contribute to managing it. Dorman and colleagues [60] have suggested the use of cognitive restructuring to address maladaptive thoughts that cause anxiety, and relaxation exercises to minimise fear while taking pills. Patterson and colleagues [62] combined cognitive behavioural therapy with swallowing therapy for head and neck cancer patients with dysphagia. Although depression and anxiety scores did not improve, patients reported that the cognitive behavioural therapy was beneficial, and several believed it contributed to their recovery and wellbeing. Including psychologists in multidisciplinary treatment teams, alongside speech pathologists and pharmacists, could be an important step towards minimising the negative psychological effects of pill dysphagia, thus improving patients’ wellbeing [35, 63].

### Strengths, Limitations and Future Directions

This study is the first to explore the impact of pill dysphagia on wellbeing across a range of indicators, incorporating participants’ insights through open-ended questions. Furthermore, it is among a limited number of studies evaluating the psychological ramifications of dysphagia within a community-based sample of older adults [47]. Although this offers advantages in terms of generalisability over previous hospital-based samples, the sampling method focused on independent community dwelling older adults and as such is the likely reason for the low prevalence of severe pill dysphagia in the current sample. The lack of participants reporting moderate to severe pill dysphagia is a considerable limitation of this study. To address this, future research could aim for larger study samples, and target subjects prone to pill dysphagia, such as residents of aged care facilities [7] or those with more complex healthcare needs. Such efforts would show whether intensified pill dysphagia drives distinct wellbeing variations.

This study adds to the literature utilising the PILL-5 scale [41]; it is only the second known study to do so. The PILL-5 provided an easy-to-use measure of pill dysphagia ideal for online self-report surveys [57]. It demonstrated good internal consistency in the current sample, adding to evidence of its reliability. However, self-report measures of dysphagia can be inaccurate [64, 65]. They are, therefore, recommended for use in conjunction with objective assessments of swallowing difficulty [66]. Therefore, future research could incorporate both the PILL-5 and an objective measure to better evaluate participants’ ability to swallow pills. Further, acknowledging the complexity of health conditions that may be associated with swallowing difficulty, including those reported by our participants, future research should also seek to conduct a full swallowing assessment for foods, drink and pills to examine differences between those individuals with dysphagia for pills only, dysphagia for foods and liquids, but not pills, and dysphagia for foods, liquids and pills.

We also note that pill dysphagia and wellbeing could have an inverse or bidirectional relationship, with poor wellbeing affecting swallowing ability. Anxiety, for example, can cause muscle tension and inhibit saliva flow [67]. However, the cross-sectional design of this study means that causality and direction of influence cannot be inferred from the quantitative results. This limitation presents opportunities for future longitudinal research examining changes in wellbeing after treatment for pill dysphagia.

## Conclusion

Pill dysphagia poses a prevalent and consequential issue for older adults, impacting their physical wellbeing significantly. This study offers novel insights into the consequences of pill dysphagia for overall wellbeing. Participants, reflecting community dwelling older adults with self-reported difficulty swallowing pills, reported physical, psychological, and practical challenges arising from their condition, leading to anxiety and difficulties in effectively managing symptoms. Notably, no direct association was found between difficulty swallowing pills and negative affect in our sample. Further large-scale studies are warranted to explore hedonic and eudemonic wellbeing among individuals with more severe pill dysphagia and examine the interplay of pill dysphagia with difficulty swallowing foods and liquids more broadly. The current findings underscore the need to improve resources to support patients with pill dysphagia. These resources should include appropriately formulated medications, education on the dangers associated with pill modification, formulation aids, and a collaborative multidisciplinary approach involving speech pathologists, psychologists, and pharmacists. Such comprehensive measures are crucial for addressing the multifaceted challenges posed by pill dysphagia and fostering the overall wellbeing of affected individuals.

## Data Availability

The data that support the findings of this study are available from the authors, upon reasonable request.
